# Optomagnetic plasmonic nanocircuits†

**DOI:** 10.1039/c9na00351g

**Published:** 2019-06-25

**Authors:** Zahraa Al-Baiaty, Benjamin P. Cumming, Xiaosong Gan, Min Gu

**Affiliations:** Centre for Micro-Photonics, Faculty of Science, Engineering and Technology, Swinburne University of Technology Hawthorn Victoria 3122 Australia zalbaiaty16@gmail.com; Laboratory of Artificial-Intelligence Nanophotonics, School of Science, RMIT University Melbourne 3001 Australia; Department of Laser and Opto-Electronic Engineering, University of Technology Baghdad 10011 Iraq; Centre for Artificial-Intelligence Nanophotonics, School of Optical-Electrical and Computer Engineering, University of Shanghai for Science and Technology Shanghai 200093 China

## Abstract

The coupling between solid-state quantum emitters and nanoplasmonic waveguides is essential for the realization of integrated circuits for various quantum information processing protocols, communication, and sensing. Such applications benefit from a feasible, scalable and low loss fabrication method as well as efficient coupling to nanoscale waveguides. Here, we demonstrate optomagnetic plasmonic nanocircuitry for guiding, routing and processing the readout of electron spins of nitrogen vacancy centres. This optimized method for the realization of highly efficient and ultracompact plasmonic circuitry is based on enhancing the plasmon propagation length and improving the coupling efficiency. Our results show 5 times enhancement in the plasmon propagation length using (3-mercaptopropyl)trimethoxysilane (MPTMS) and 5.2 times improvement in the coupling efficiency by introducing a grating coupler, and these enable the design of more complicated nanoplasmonic circuitries for quantum information processing. The integration of efficient plasmonic circuitry with the excellent spin properties of nitrogen vacancy centres can potentially be utilized to extend the applications of nanodiamonds and yield a great platform for the realization of on-chip quantum information networks.

## Abstract 2

Optomagnetic plasmonic nanocircuitry for directing and processing the readout of electron spins of nitrogen vacancy centres in nanodiamonds.

## Graphical abstract

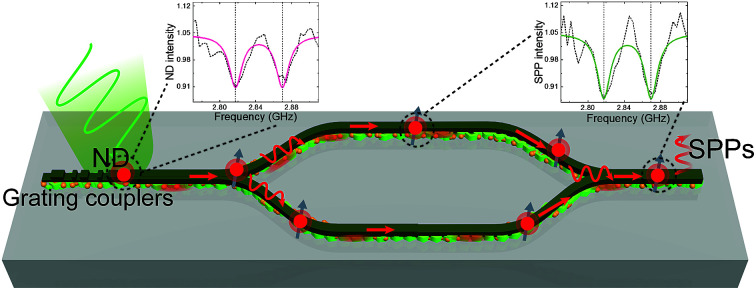

## Introduction

Nanoscale waveguide-based surface plasmon polaritons (SPPs) are imperative for the ultimate miniaturization of photonic circuitries beyond the diffraction limit. Such circuitries have a wide range of potential applications, in areas such as quantum information, optical communication, and sensing. Consequently, a number of impressive demonstrations have already been made for SPP propagation along various types of plasmonic waveguides.^1–5^ Nonetheless, realizing such devices undoubtedly depends on chip integration. The latter calls for the development of compact optical sources capable of directing some of its emission into a single mode waveguide.^6,7^ It has been shown that controlled routing of single plasmons in a plasmonic circuit comprising a single photon source, nitrogen vacancy (NV) centres in nanodiamonds (NDs), and plasmonic waveguides is possible,^2,4^ which may lead to the construction of a complete quantum plasmonic circuit.

**Fig. 1:**
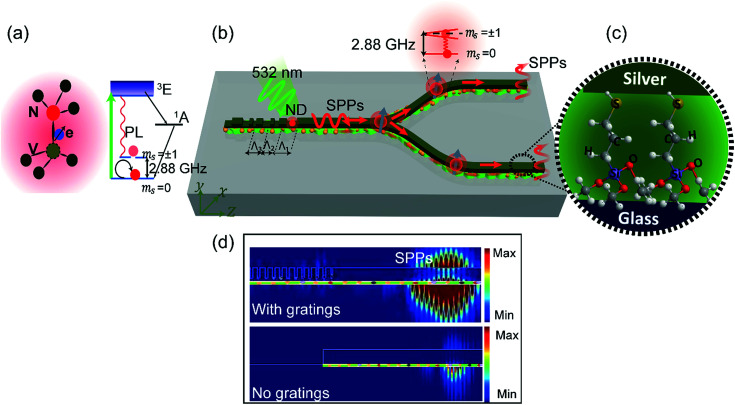
(a) Nitrogen vacancy centre in nanodiamonds. The left side shows the atomic structure of the NV defect in the diamond, and the right side shows the energy level structure of the NV centre. (b) Schematic illustration of the lithographic plasmonic circuitry which is able to control and process the readout of the electron spins of NV centres in NDs. (c) Zoomed image of the MPTMS adhesion layer. (d) An optimized grating coupler for high coupling efficiency and directionality of the NV centre broad emission in the plasmonic circuitry.

Furthermore, nanodiamonds are particularly interesting due to their unique properties of NV centre electron spin, which can be optically read out and initialised by a technique known as optically detected magnetic resonance (ODMR).^8–10^ The spins associated with the NV centre defects in diamond, Fig. 1a, have been identified as promising spin qubits for the practical realization of solid-state spin based quantum information processing^9,11,12^ and nanoscale magnetometry schemes^13–16^ at room temperature. In these configurations, the quantum information can be stored in the electron spin states of the NV centre while quantum logic can be achieved by modulating the photoluminescence intensity *via* the ODMR technique.^17,18^ Nonetheless, the ODMR technique is challenging due to the long radiative lifetime and low collection efficiency of photons emitted by the NV centres.^19–21^ An efficient approach to overcome this issue is by coupling the ND emission to plasmonic modes.^22–24^

In particular, coupling the readout of the electron spins of NV centres in NDs to plasmonic modes can be employed for realizing on-chip quantum information processing where the plasmonic waveguides can be used as networks to guide, process and transfer the spin quantum information along chip-scale devices. So far, experimental investigations on the readout of the ODMR signal in propagating SPPs have only focused on the study of chemically grown silver nanowires^25^ which are difficult to integrate into circuity. Conversely, lithographically fabricated plasmonic nanowires allow controllable circuitry design, but show significantly higher losses than chemically grown nanowires^26^ due to damping by the metallic adhesion layer^27–30^ which is required to guarantee good bonding between the plasmonic structure and the glass surface.^31,32^ Even with recent demonstrations of plasmonic nanocircuitry with NDs,^2,33,34^ compact devices with a high coupling efficiency and low propagation loss remain elusive.

In this report, we develop compact optomagnetic plasmonic nanocircuitry for processing the ODMR signals of NV centres. We demonstrate that the ODMR spin state readouts can be coupled to plasmonic modes of lithographically fabricated nanowires (NWs), and passed through plasmonic elements such as beam splitters (Fig. 1b), which are essential elements needed to form quantum networks. To enhance the propagation length of the SPPs, we employ a lithographic fabrication method based on (3-mercaptopropyl)trimethoxysilane (MPTMS) as an adhesion layer (Fig. 1c). Additionally, to enhance the coupling efficiency and achieve high directionality, and to collect the broad emission spectrum of the NV centres, the plasmonic NWs were integrated with on-chip optimized grating couplers (Fig. 1d). Together, this approach enables substantial enhancement in the performance of plasmonic NWs that can be scaled to more complicated multifunctional circuitries and leveraged for quantum information technology.

## Experimental section

### Fabrication method using MPTMS

The arrays of plasmonic structures used in this experiment were fabricated on a glass substrate. The substrate was cleaned in Piranha solution to remove metals and organic contaminants. The glass was immersed in a freshly prepared Piranha solution composed of sulphuric acid (H_2_SO_4_) and hydrogen peroxide (H_2_O_2_) (3 : 1) for 30 min at 70 °C. To functionalize the glass with MPTMS, we optimized the method proposed by Goss *et al.*^35^ to improve the performance of the adhesion layer but for silver nanostructures instead of gold. In the hydroxylation process, the glass was placed on a hot plate at 100 °C for 10 min to leave –OH groups on the surface of the glass. Meanwhile, a silanization solution was prepared by adding 5 g of MPTMS to a solution of 5 g of H_2_O_2_ and 200 g of 2-propanol (IPA). After the solution was heated to boiling, the dried glass was immersed in it for 10 min, then rinsed with IPA and dried with nitrogen. The glass was then cured by placing it on a hot plate at 110 °C for 10 min. The procedure of immersing in MPTMS solution, rinsing and curing was repeated an extra two times to ensure high probability of covalent Si–O–Si bonds on the glass surface.

Arrays of nanowires and splitter patterns were then defined using an electron beam lithography (EBL) system (Raith 150) at an accelerating voltage of 10 kV, a beam aperture size of 10 μm, a working distance of 10 mm and a beam current of 0.017 nA. Electron beam evaporation is used to obtain a thin Ag film with a final thickness of 70 nm. The deposition was run at a low-pressure of ≈ 2 − 4 × 10^−7^ Torr with a 1 Å s^−1^ deposition rate. The deposition processes were separated by 15 min of rest time to allow the sample to cool and avoid damaging the molecular linker. The lift off process was carried out by soaking the sample in acetone for 3 h, followed by sonication bath in acetone for 3–5 min. Finally, the sample was rinsed with IPA and dried with nitrogen.

### Preparing the sample for the ODMR measurements

For the ODMR experiments, a glass substrate having a gold antenna in the shape of two triangles connected in the middle by a ≈20 μm gold micro-antenna (inset of Fig. S1†) was fabricated using electron beam evaporation of 25 nm Cr and 75 nm Au, to allow microwave signals to pass through. Microwave signals from a microwave generator (Agilent N5183A MXG), between −18 dBm and −20 dBm, amplified using an Ophir 5181, passed through the gold micro-antenna from one end to an oscilloscope on the other end. For the sake of precise alignment in the positioning of the plasmonic structures so that they are in close proximity to the gold micro-antenna, we utilize a previously captured scanning electron microscope image and depend on the coordinate of three global marks identified on the structures of the antenna. The ODMR experiments are performed by combining optical and microwave excitation integrated with optical detection (Fig. S1†). To excite NV electron spins from the *m*_s_ = 0 state to the *m*_s_ = ±1 states, the excitation laser is switched on while applying a resonant microwave field. Simultaneously, the emission from the NV centre and the scattered SPP signal at the output end of the nanowire are collected through a high NA objective and directed towards an electron multiplying charge-coupled device (EMCCD) for imaging.

## Results and discussion

### Optimization of the plasmonic NWs

Our device geometry, shown in Fig. 1b, has been optimized and investigated both theoretically and experimentally toward efficient on-chip ODMR detection of NV centre spin states. Fig. 1b depicts the schematic of a plasmonic beam splitter coupled to a ND that can be excited with a 532 nm CW laser. The emitted photons from the NDs are coupled to the plasmonic modes and propagate along the device. The optimization methods have been well investigated for the purpose of achieving a longer plasmon propagation length (*L*_sp_) and a higher coupling efficiency (*η*). Hence, the net coefficient in the NW (*η*_NW_) is defined as *η*_NW_ = exp(−(1/*L*_sp_)*z*) × *η*, where *z* is the local position along the NW.

**Fig. 2:**
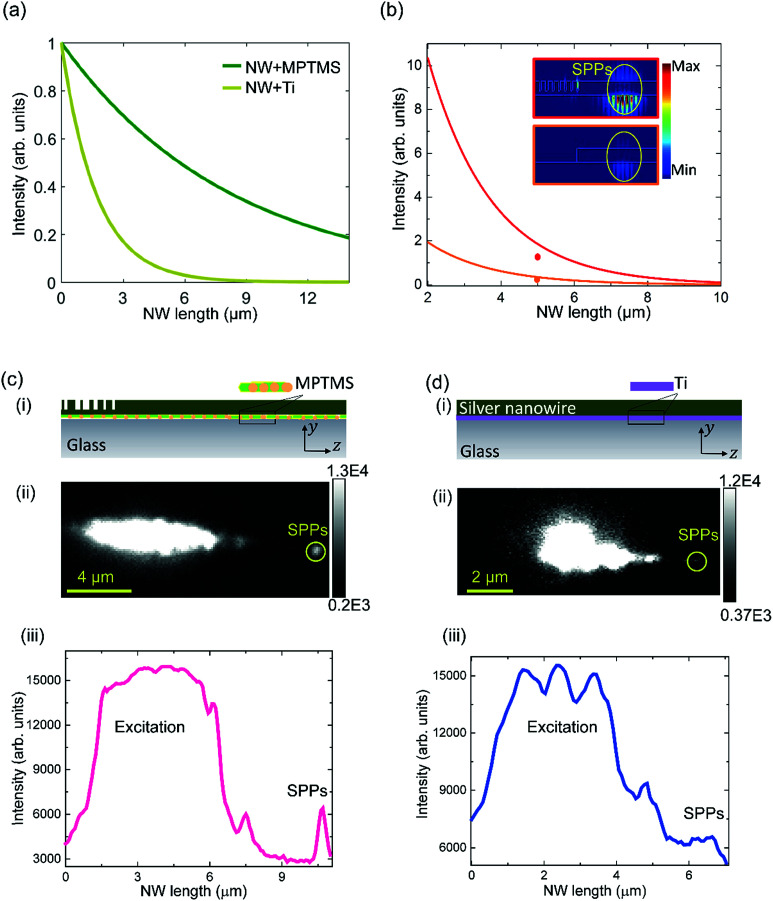
Characterisation of efficient lithographic NWs for on-chip plasmonic circuitry. (a) Simulation of NWs showing the measured *I*_sp_ as a function of the NW length for NWs deposited on either a 2 nm Ti adhesion layer or a MPTMS adhesion layer. (b) *I*_sp_ as a function of the NW length for two different coupling schemes. The dots in (b) present the experimentally obtained results. (c) Experimentally measured SPPs along NW-OGCs fabricated on the MPTMS pre-treated glass substrate. (d) Experimentally measured bare NW fabricated on a 2 nm Ti adhesion layer. Schematic illustration of the systems in (c) and (d) are shown in c(i) and d(i), respectively. The respective photon emission maps and the intensity profiles are shown in c(ii) and d(ii) and c(iii) and d(iii), respectively.

To enhance *L*_sp_, the plasmonic structures were fabricated using the EBL method with MPTMS, which contains functional groups at both terminals of the molecule, as an adhesion layer^32,36,37^ (Fig. 1c). We numerically investigate plasmonic NWs represented by rectangular lithographic silver NWs of width *w* = 100 nm and thickness *t* = 70 nm in the *xy* plane (Fig. S2, ESI†) which feature both reasonably good propagation length and mode confinement. The propagation characteristics of the NWs based on MPTMS adhesion layers are compared with those of traditional lithographically fabricated NWs which are attached to the glass substrate with titanium (Ti) adhesion layers. Simulations for various NW lengths, 2 μm to 14 μm, are carried out (Fig. 2a). By fitting the results to an exponential decay function as:

1 *I*(*z*) = *I*_0_ × e^−*z*/*L*_sp_^

where *I*_0_ is the initial intensity, an enhancement in the propagation length for NWs supported with the MPTMS adhesion layer is observed compared with Ti-based systems. Our simulation results shown in Fig. 2a predict an *L*_sp_ of 8.3 μm for the NW on the MPTMS adhesion layer which is a factor of 5 times longer than that of the traditional Ti-based system.

In the experiment, NWs were fabricated using EBL onto either a 2 nm Ti or MPTMS functionalized glass substrate. MPTMS forms a thin monolayer^37–39^ and has a refractive index comparable with the refractive index of the glass substrate;^36,37^ thus the precise thickness of MPTMS does not significantly influence the properties of the plasmonic modes. The NWs are then optically characterized using a confocal and widefield fluorescence microscope system using the experimental setup shown in (Fig. S1†). We measure the propagation characteristics of the NWs based on different adhesion layers by using eqn (1) and by comparing the ratio of the output photons emerging from the NW end to the input excitation intensity, *i.e.* the *I*_sp_, for different NWs (more details are available in the ESI†).

Normalized by the total energy input into the SPPs, our results show that using MPTMS as an adhesion layer can lead to an 86% enhancement in the *I*_sp_ (Table S1†) compared with the Ti adhesion layer. These results show that adding a thin metallic adhesion layer of Ti limits the *L*_sp_ of the SPPs. For other metallic adhesion layers such as chromium, the losses could be worse due to stronger absorption.^27^

To make maximum use of on-chip SPP routing, light must be coupled efficiently to the guided plasmonic modes; thus we introduced a chirp into the grating period and groove length. By varying the grating parameters, the strength of the scattered intensity from the grating can be controlled. Numerical simulations and optimization of the nanograting couplers for efficient excitation of the SPPs at an Ag–SiO_2_ interface, and for operation at a wavelength of 700 nm are carried out (Fig. S3–S6, ESI†). The upper inset of Fig. 2b shows the SPP power flow when the optimized grating couplers are introduced. For the purpose of comparison, we also consider propagating SPPs along a bare NW (lower inset of Fig. 2b). The coupling efficiency in the NW integrated with the optimized grating coupler (NW-OGC) system is measured (by monitoring the values of *I*_sp_) and found to be considerably larger than that of SPPs excited *via* end coupling (*i.e.* no grating). The simulation results (Fig. 2b) indicate an enhancement in *η* by a factor of 5.2 with respect to that of end coupling measured at 2 μm propagation distance away from the excitation source.

To determine the coupling efficiency, we experimentally measured the SPP excitation on the integrated NW-OGC system and extracted the *η* from the wide field fluorescence images obtained using the EMCCD camera. The experimental results show that a 4.0 ± 0.5 times enhancement in *η* is possible when the optimized grating couplers are employed in the system compared with end coupling. These results are in good agreement with the simulation results (Table S1†) indicating that the NW-OGC system is a significantly more efficient coupling scheme that allows for more advanced device architectures. It should be pointed out that for the purpose of comparing coupling efficiencies with and without the grating, we use the same adhesion layer (Ti) for the two cases so that any propagation loss cancels out.

To illustrate the overall performance improvement by combining the couplers and MPTMS adhesion layer, we performed experimental characterisation of the NW-OGC systems fabricated on a MPTMS pre-treated glass substrate (shown schematically in Fig. 2c(i)). The results were compared to lithographic NWs fabricated on a thin Ti adhesion layer and excited by end coupling (shown schematically in Fig. 2d(i)). We study the output of the coupling signal and the excitation signal along the nanowire obtained from the optical image. Clearly, an enhancement in the SPP propagation signal has been achieved when both a low loss adhesion layer and an efficient coupling scheme are used together (Fig. 2c(ii) and (iii)) compared with a bare NW on a Ti adhesion layer (Fig. 2d(ii) and (iii)). A comparison of the two systems shown in Fig. 2c and d indicates that a 6 times enhancement in *η*_NW_ can be obtained experimentally (8 times enhancement obtained from numerical simulations).

### Surface plasmon detected magnetic resonance

**Fig. 3:**
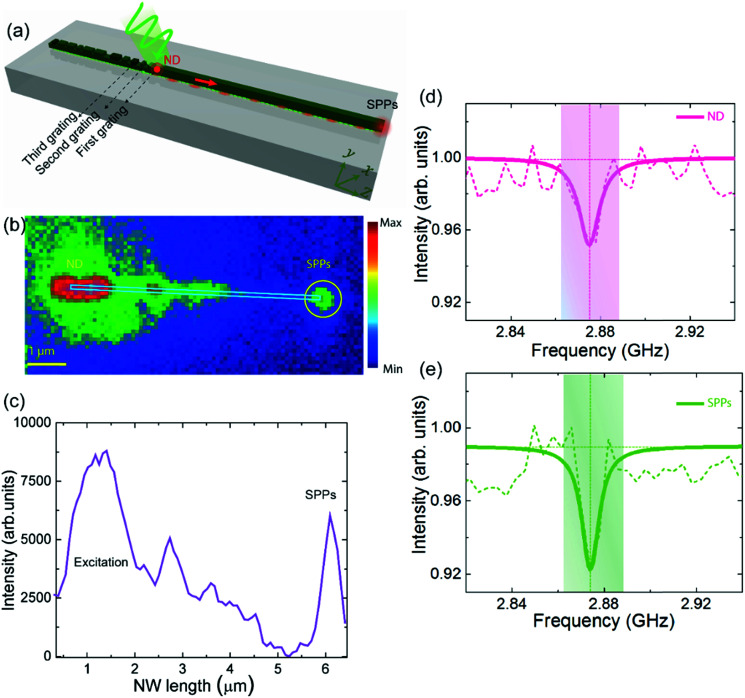
(a) Schematic of the NW-OGC system based on a MPTMS adhesion layer coupled to the NV centre in NDs showing a map of the variable displacement of the ND. The corresponding photon emission map and intensity profile are shown in (b and c), respectively. Measurement of the NV spin state readout in a ND coupled NW-OGC system showing a reduction in the photoluminescence intensity in ND emission (d) and a reduction in the SPP scattering strength (e).

Here we assess the suitability of the optimized NW-OGC system for integrated solid-state quantum systems. We probe the coupling efficiency when the position of the emitter is moved with respect to the *y* and *z* axes of each groove in the gratings (Fig. 3a). The coupling efficiency of an emitter to the NW-OGC system can reach 90% (Fig. S7a†). Our simulation results show that we can enhance the coupling efficiency by up to 12.4 times compared to the case when ND emission is coupled to a bare NW. However, this coupling is reduced drastically when the emitter is placed at the second or third element of the grating (Fig. S7b and c†). Furthermore, the influence of the vertical displacement of the emitter on coupling shows that the highest coupling can be achieved when the emitter is located closer to the metallic surface (red lines in Fig. S7†) due to the induced Purcell enhancement in the emission of the nanoemitters.^23,40–44^ Accordingly, we designed the first two grating elements such that it can host only one ND of 70 nm in size for later investigation in the experimental work. Our numerical analysis shows that exploiting optimized grating couplers can enable efficient collection of emitted photons and direct them to the nanocircuits.

To experimentally demonstrate the coupling efficiency of the system presented in Fig. 3a, an aqueous solution of NDs with an average size of 70 nm was sonicated before being spin-coated on a glass substrate containing arrays of lithographically fabricated NW-OGC. Widefield fluorescence and white light imaging are used to locate a ND located at the first or second grating element close to the edge of the NW. An EMCCD camera image shows coupling between a ND and NW-OGC and subsequent evidence of SPP emission at the far end of a 5 μm long silver nanowire (Fig. 3b) when the ND is excited with a 532 nm CW green laser. The emission intensity profile is plotted as a function of the distance along the NW-OGC as shown in Fig. 3c. By comparing the ratio of the output SPP intensity to the input excitation intensity (including all the intensities measured at the ND position), our experimental results predict 68.3 ± 2.5% of the total power being coupled to propagating SPPs supported by the NW-OGC system.

The NW-OGC system we proposed in this work, which utilizes efficient coupling and low loss SPPs, is efficient and significantly smaller than previously realized plasmonic structures.^2,33^ It also shows the ability to further develop subwavelength integrated on-chip plasmonic circuits that are viable for on-chip quantum information processing.

The optical readout of the spin states of the NV centres relies on fluorescence intensity measurements. We now probe our NW-OGC system when coupled to NV centres and demonstrate the conservation of the NV centre spin state readout after propagating through the circuit. We measure the spin states of the NV centres when coupled and propagated *via* SPPs along the NW-OGC attached MPTMS pre-treated glass substrates. We probe the potential of these nanowires to guide and maintain the NV centre magnetic resonance by performing ODMR measurements. The NDs are continuously pumped *via* green light whilst a microwave frequency around the zero-field resonant transition between the *m*_s_ = 0 and *m*_s_ = ±1 spin states of the NV centre was swept. At each frequency within the sweep, the strength of both the ND emission and the emission from the nanowire ends are monitored. The ODMR spectra of the ND emission (Fig. 3d) and SPP scattering (Fig. 3e) are fitted with the Lorentzian function to determine the quality of resonance before and after propagating along the NW-OGCs.

We observe a reduction in the SPP scattering intensity at a frequency of ≈2.875 GHz similar to the reduction observed in the ND emission. Furthermore, the linewidth of the resonance in the SPP spectra matches that in the ODMR spectra with an average value of 10 MHz. Furthermore, a change of 39.8% in the contrast between the SPP scattering signal and ND emission (*R*_cSPP_), where *R*_cSPP_% = (*I*_baseline_ − *I*_dip_)/*I*_baseline_, is observed when the ND emission is coupled to and propagated along the NW-OGC system. These results reveal that the NV centre spin readout can be preserved and propagated along lithographically defined SPPs in a similar trend to what we observed before.^25^ Also, we have observed an improvement in the spin contrast due to an improvement in the signal to noise ratio in the ODMR signal at the far end of the NW away from the excitation point (where the luminescence of NV centres and autofluorescence of the glass substrate overlap). This contrast in NW-OGC is higher even when compared to coupling to NW only (Fig. S8†); the latter enhancement in contrast in the SPP signal can be attributed to both noise filtering and directional effect of the NWs.

### Optomagnetic plasmonic circuitry

**Fig. 4:**
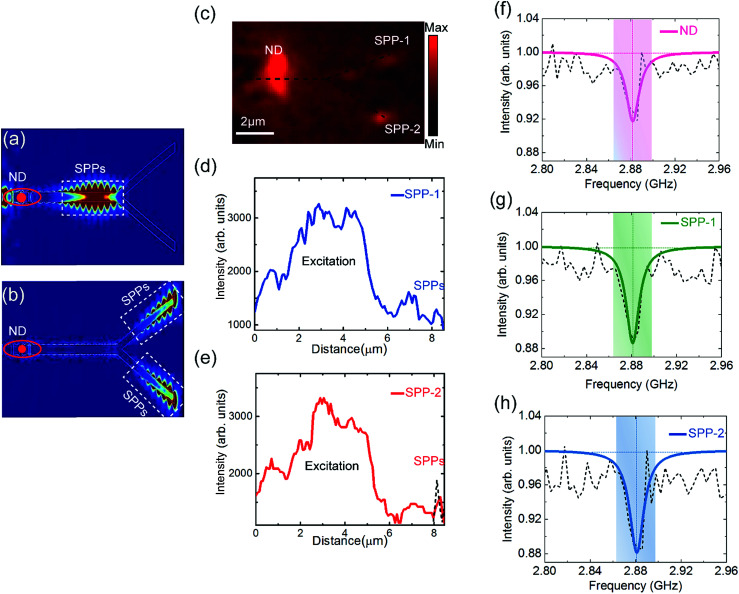
(a and b) The time domain snapshot observed in 2D view from the FDTD simulation of bound SPP modes propagating along the PS-OGC system showing the SPP power flow before and after being split. (c) EMCCD camera image of the plasmonic beam splitter when a ND is excited and the resulting emission at the far end of the splitter ports, confirming the coupling from the NV centre to the plasmonic mode. (d and e) The intensity profile for the upper and lower ports, respectively, in the plasmonic splitter–ND coupled system. The black dashed line in (e) shows the sum of the SPP signal intensity at both ends of the splitter. Measurement of the NV spin state readout when coupled to the plasmonic splitter proves the conservation of the ODMR signal after propagating along the splitter output ports. (f) ODMR signal in ND emission, and (g and h) ODMR signal in the upper and lower output ports of the splitter, respectively.

In this section we demonstrate that optomagnetic plasmonic circuitry (Fig. 1b) can be used to guide, split and process the ODMR signal from the NV centre into different arms. In particular we demonstrate propagation through a plasmonic splitter as it is an important component for routing, extracting information and on-chip realization of quantum optical networks. The circuit shown in Fig. 1b is numerically simulated (Fig. 4a and b) to determine the resulting net coefficient (*η*_all_) by reading the strength of the power flow of propagating SPP intensity along the circuitry. We derive a simple expression for *η*_all_ as *η*_all_ = exp(−(1/*L*_sp_)*z*) × *η* × *η*_splitter_, where *η*_splitter_ is the splitter efficiency defined as the relative SPP intensity at the end of the splitter ports (*I*_a_ and *I*_b_), as indicated in Fig. S9a,† expressed as a fraction of the relative SPP intensity at the beginning of the splitter (*I*_c_). The design characterisation of the plasmonic circuitry as a function of the splitter length and offset distance is shown in Fig. S9b and c.† Numerical analysis reveals that the *η*_all_ for the demonstrated plasmonic circuitry reaches up to 29.5% obtained by integrating the overall efficiency of each component.

The coefficient of the system is experimentally measured. The unsaturated photon emission map (Fig. 4c) and the intensity profile along the splitter ports (*I*_spp-1_ and *I*_spp-2_ shown in Fig. 4d and e, respectively) are used to estimate the experimental *η*_all_ by plotting a line cut off intensity extracted from the wide field fluorescence images starting from the excitation spot. Since in the experiment we can only read the excitation and out-coupling intensities, we depend on them for estimating *η*_all_. Our experimental results show that a *η*_all_ of 27.7 ± 2.3% can be obtained in the studied plasmonic splitter integrated with the optimized grating coupler (PS-OGC) system.

Furthermore, we investigated the ability of the demonstrated plasmonic circuitry to guide and process the NV centre spin state readouts when coupled and propagated through the circuit. We performed ODMR measurements and monitored the emission from the ND in the excitation area and the scattered SPPs at the far ends of the splitter. We observed the characteristics of the ODMR spectra in the ND emission and in the SPP scattering strength with a reduction in the SPP intensity at an applied microwave frequency of 2.88 GHz, matching with the photoluminescence spectra of the NV centre. In addition, the line width of magnetic resonance remains consistent at ≈14 MHz for the ND and SPPs at both splitter ends. We defined the change in the contrast between the SPP scattering signal at both ends of the splitter and the ND emission as ((*R*_cSPP-1_ − *R*_cND_)/*R*_cND_) and ((*R*_cSPP-2_ − *R*_cND_)/*R*_cND_), where *R*_cSPP-1_ and *R*_cSPP-2_ represent the ODMR contrast in the SPPs in the upper and lower ports of the splitter, respectively. Our results show that the change in the contrast between the SPP signals and ND emission is improved by a factor of 38% and 40% in the upper and lower splitter ports, respectively. These attributes mark a critical point in utilizing the NV centres for various emerging quantum technologies.

## Conclusions

Realization of functional quantum plasmonic circuitries requires solving a number of problems, such as plasmonic losses and efficient coupling between nanoscale elements. One area that depends on a solution to these challenges is the coupling of quantum emitters to plasmonic devices. Such systems can be used for the optical initialization and readout of electron spins, and can be leveraged for solid-state quantum technologies.

We demonstrate that the ODMR readout of the electron spins of NV centres can be coupled to plasmonic modes of lithographically fabricated NWs. Our approach for the development and implementation of efficient NWs is based on the optimization of the nanofabrication method. This approach makes the performance of the lithography-based silver NWs more reliable and efficient for nanoscale plasmonic circuitry design, and it can potentially be employed for fabricating other waveguides, such as those in ref. 45 and 46. To improve the coupling efficiency, we have introduced optimized chirped grating couplers to the lithographic NWs. Particularly, we show 5 times enhancement in the plasmon propagation length using MPTMS, and 5.2 times improvement in the coupling efficiency; together these improvements enable the design of more sophisticated networks.

We demonstrate on-chip optomagnetic plasmonic circuitry based on efficient NWs for directing and processing the spin state readouts of NV centres. Our experimental results show the conservation of the NV centre spin state readouts after propagation and splitting through the circuitry. We investigate the ODMR signals along the nanoplasmonic splitter to prove the concept. Nonetheless, our promising simulation results (Fig. S10†) show that this method can be utilized to extend the functionality of such devices for advanced signal analysis and processing, leading the way to exploring the benefit of NDs for practical on-chip quantum networks.

## Conflicts of interest

The authors declare that they have no conflict of interest.

## Supplementary Material

NA-001-C9NA00351G-s001
