# Supplementary material for: Optomagnetic plasmonic nanocircuits
Source: Nanoscale Adv. 2019 Jun 25;1(8):3131–8. doi: 10.1039/c9na00351g (PMC9418874; doi:10.1039/c9na00351g)
Supplement: NA-001-C9NA00351G-s001 [file NA-001-C9NA00351G-s001.pdf]

Electronic Supplementary Material (ESI) for Nanoscale Advances.
This journal is © The Royal Society of Chemistry 2019

Optomagnetic plasmonic nanocircuits

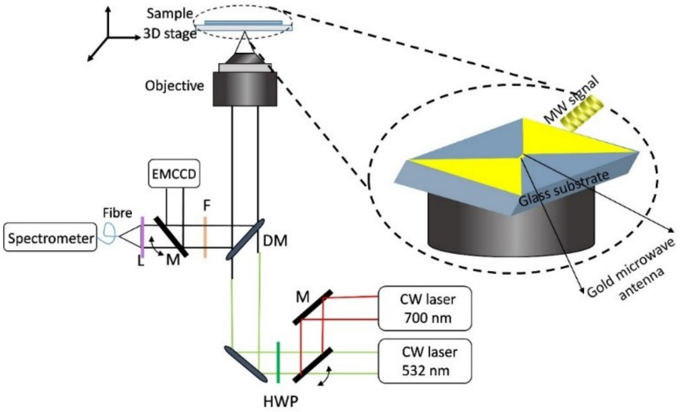

Figure S1. **Experimental setup**. A confocal microscope system with a 532nm/700 nm laser excitation wavelength. **M**-mirror, **HWP**-half waveplate, **F**-fluorescence filters for photon wavelength lies between 647 and 785 nm, **L** focusing lens and **DM**- dichroic mirror.

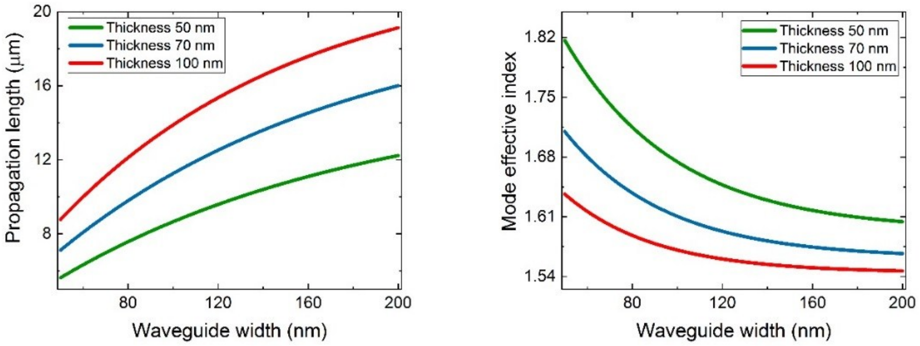

Figure S2. (**a**) Plasmon propagation length, and (**b**) Mode effective index for TM mode supported by silver NW at 700 nm plotted as a function of the width of the plasmonic waveguide for different thickness.

Supporting information

Optimization of the plasmon propagation length

We investigate plasmonic waveguide represented by a rectangular lithographic silver NW of width w=100 nm and thickness t=70 nm in the xy plane, and with optical constants being adopted from experimental data by John and Christy [1]. The FDTD numerical simulations implemented using Lumerical, with artificial absorbing boundaries surrounding the computational domain, are used to investigate the effect of the MPTMS or Ti as an adhesion layer on plasmon propagation length. Two identical NWs supported with a 2 nm Ti adhesion layer, or a 2 nm MPTMS adhesion layer are investigated. The intensity (I_{sp}) of the propagating SPPs signal along the NWs when excited with a 700 nm for both types of samples are measured by placing a set of power monitors (detectors) in the transverse xy plane at the far edge of the NWs from the excitation area. The first monitor is set at z=2 µm from the edge of the NW in order to monitor only the guided SPPs signal and to eliminate the residual effect of the excitation source. Using a 700 nm excitation source we excite SPPs at the Ag-SiO_2 interface to be guided along NWs with varying length. By fitting the results in Fig. 2a to an exponential decay function as Equ. (1) The effect of the adhesion layers (Ti or MPTMS) on L_{sp} of propagating surface plasmons is found.

In the experiment, light from a CW tunable Ti:Sapphire laser with a wavelength of 700 nm is linearly polarized and adjusted with a half waveplate. This laser beam is used to excite SPPs along the NW, which in turn propagate along the NW before being scattered and partially converted into free space photons at the far end of the NW. The scattered SPPs signal is collected by a microscope objective and is optically imaged onto an EMCCD camera without saturation. The scattered SPPs intensity is identified by the maximum value in the emission site (away from the excitation spot) obtained from the optical image. Consequently, a photon emission map and intensity profile along the NWs are obtained which are used later to investigate the propagation characteristics for the NWs.

Optimization of the grating design

To study the enhancement in the coupling efficiency of the optimized grating couplers, we design 14 element gratings with varying dimensions (Fig. S3 a). The period of the gratings (Λ) is varied from 295 nm to 330 nm while the groove length (l) is changed between 79 nm to 162 nm. The Λ and l for each element are designed in such a way to support propagating SPPs at Ag-SiO_2. The position and the injection angle of the excitation source are determined such that it excites SPPs propagating vertically up the Ag-SiO_2 interface, along the z-direction. Fig. S3 b&c shows FDTD simulation of NW-OGC and NW systems, respectively. It is shown that the guided mode is mainly confined within the Ag-SiO_2 interface but extend slightly into the adjacent air region. We fix the dimensions of the grating in the xy plane, we investigate the effect of

varying Λ and l and the corresponding relative I_{sp} of the propagating SPPs. For comparison, we also investigate measuring the I_{sp} of the propagating SPPs along NW only. Fig. S3 b&c is a video recording (frame capturing) obtained from the FDTD simulation by monitoring the systems in the yz plane, demonstrating the effect of introducing an optimized grating coupler for the efficient generation of the SPPs.

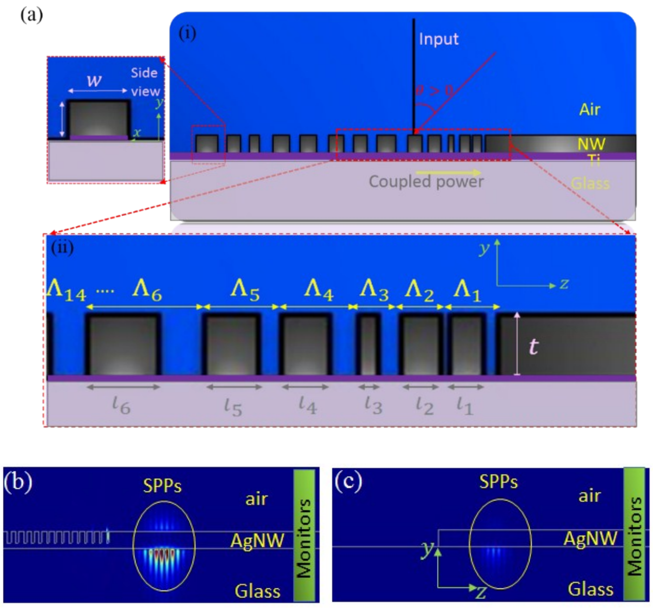

Figure S3. **FDTD simulation of the studied optimized grating coupler**. (**a**) Schematic of the optimized grating design. (**i**) Ilustration of the optimized grating couplers. Inset, close-up of a single groove showing the sideview dimensions. (**ii**) Part of the optimized gratings consist of multiple blocks of varied periodicity and length. (**b&c**) Illustrate the importance of the used grating couplers in enhancing the strength of the coupled SPPs compared with a bare NW.

1- Dependence on the geometry variations

Here we present an investigation on the effect of modifying the grating dimensions on the efficiency of the grating. As the efficiency of the grating coupler is limited by its directionality. Here, the coupling efficiency is investigated under the implementation of a thin Ag film between the grating elements. Fig. S4 shows a significant enhancement can be achieved when we fill the area between the grooves with a 23 nm thick film of Ag. The latter can be attributed to the improvement in the directionality of the grating couplers which is a function of the film thickness i.e. the etch depth of the grating. To show the sensitivity of our optimized grating couplers relative to the percentage changes in their geometries, I_{sp} is

measured over 14 μm NW length integrated with grating couplers and by reading the measurements from the power monitors. Fig. S5 shows the significant of the optimized grating in generating the efficient propagating SPPs signal even when all the geometries are reduced by 10 % from the optimal design (blue curve in Fig. S5). These results emphasis the capability of the proposed grating couplers to produce a high coupling efficiency even when small variation in the geometry may occur during the lithographic fabrication process.

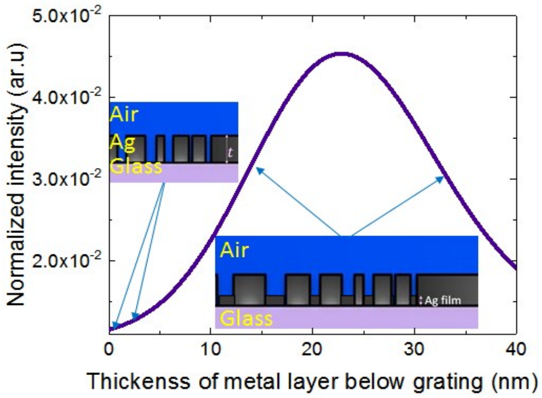

Figure S4. **Optimization of the grating couplers for efficient excitation of the SPPs**. The efficiency of the optimized grating coupler as a function of the thickness of the Ag film. The insets show illustrations when Ag film at the optimum/zero thickness.

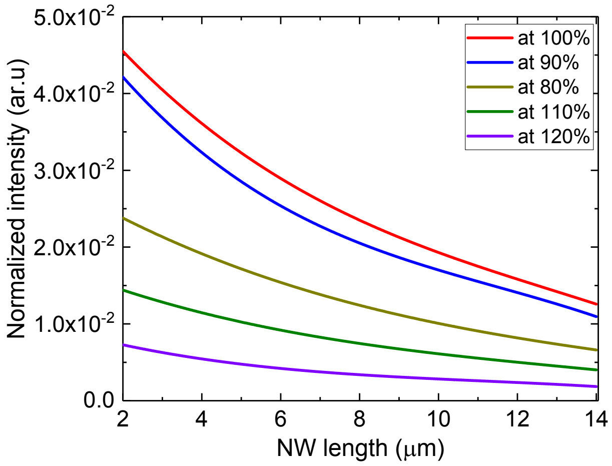

Figure S5. **Validating the optimized grating couplers design**. Normalized intensity measured along the NWs for varied percentage change in the grating coupler dimensions.

(a)

(b)

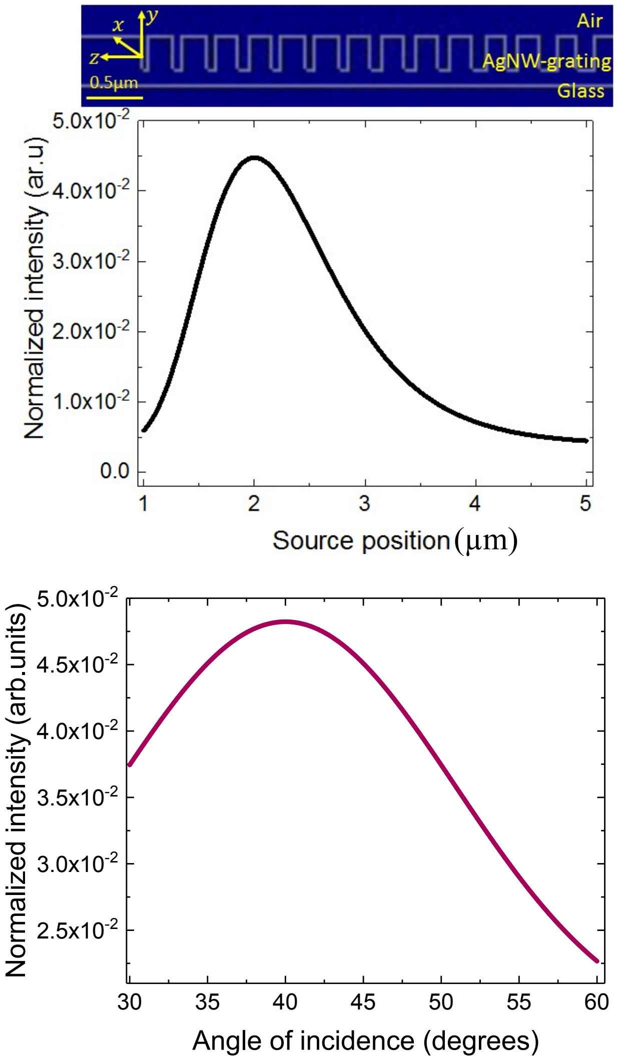

Figure S6. The coupling efficiency of the optimized gratings as afunction of source position in (**a**) and angle of incident beam in (**b**). Results show the sensitivity of our optimized grating couplers to small changes in the coupling position while there is no significant changes in the coupling efficinecy when the anlge of the incident beam is varied. The inset in (**a**) shows the studied coupling areas for the respective positions of the excitation source.

1- Dependence on the source position and the incident angle

The location of the source is set in such a way to excite and to propagate SPPs along the Ag-SiO_2 interface, i.e. along the z-direction. For maximum coupling efficiency, we place the source in the middle of the NW-grating, at x=0. We investigate the detailed coupling mechanisms when the source location is moved along the grating coupling area, i.e. along z-direction. We study five coupling regions, as indicated in the inset of Fig. S6 a. Fig. S6 a shows the sensitivity of the grating to the variations in the source position. We find moving the location of the source by 0.5 µm from the optimal location resulted a reduction in the efficiency by a factor of 1.5.

Further, we test the sensitivity of our optimized grating as a function of the angle of incidence. The results in Fig. S6 b show that the angle of the incident beam can be varied by $\pm$5 degree without significantly reducing the efficiency of our optimized grating couplers. These results confirm the flexibility of the proposed design for the grating couplers.

Table S1: Summarizing simulation and experimental results.

	Simulation results	Experimental results
Adhesion layer	$I_{spp}/I_{excitation}]_{MPTMS/Ti}$= 2.20	$I_{spp}/I_{excitation}]_{MPTMS/Ti}$=1.86±0.28
Coupling scheme	$I_{spp}/I_{excitation}]_{grating/no\ graing}$ =5.2	$I_{spp}/I_{excitation}]_{grating/no\ graing}$ = 4.0±0.5
Internal source	$I_{spp}/I_{excitation}]_{grating/no\ graing}]_{Dipole}$ =12.40	$I_{spp}/I_{excitation}]_{grating/no\ graing}]_{Dipole}$ = 12.19±0.37

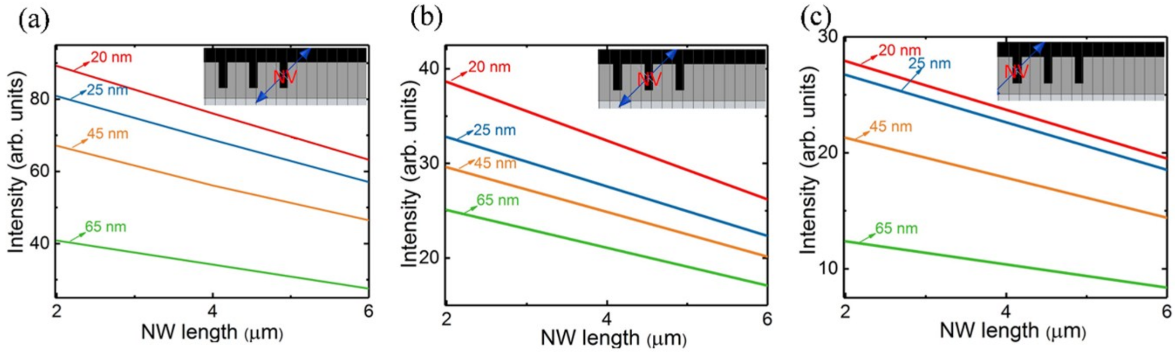

Figure S7: (a-c) Characterisation of the coupling between a dipole emitter and the NW-OGC when the dipole is place in the first, second, or third grating, respectively, as a function of varied coupling distance.

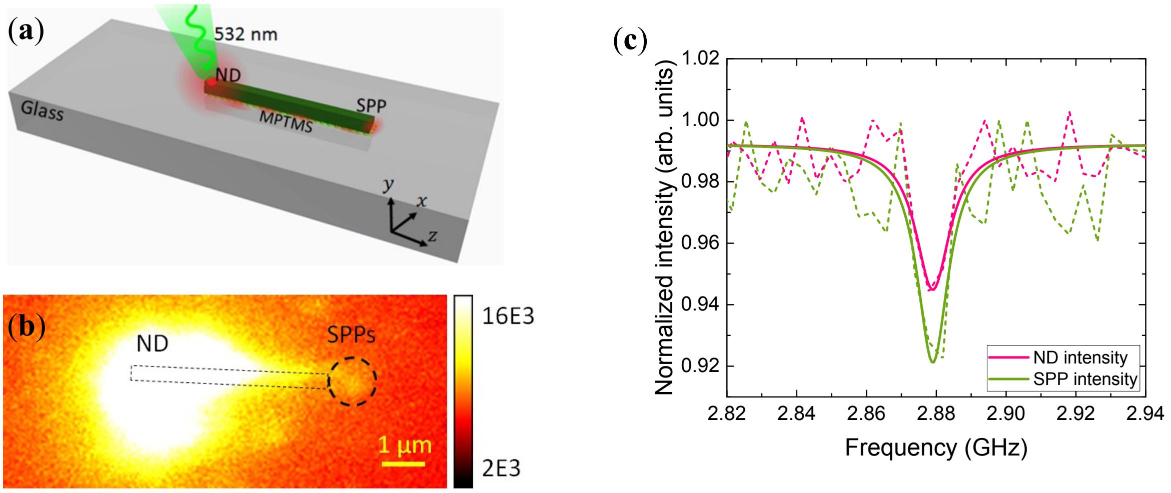

Figure S8: (**a**) Schematic figure of a lithographic NW-ND system. (**b**) The photon emission map of the studied system. (**c**) ODMR signal in the NV centre emission (pink lines) and ODMR signal in the SPPs intensity (green lines).

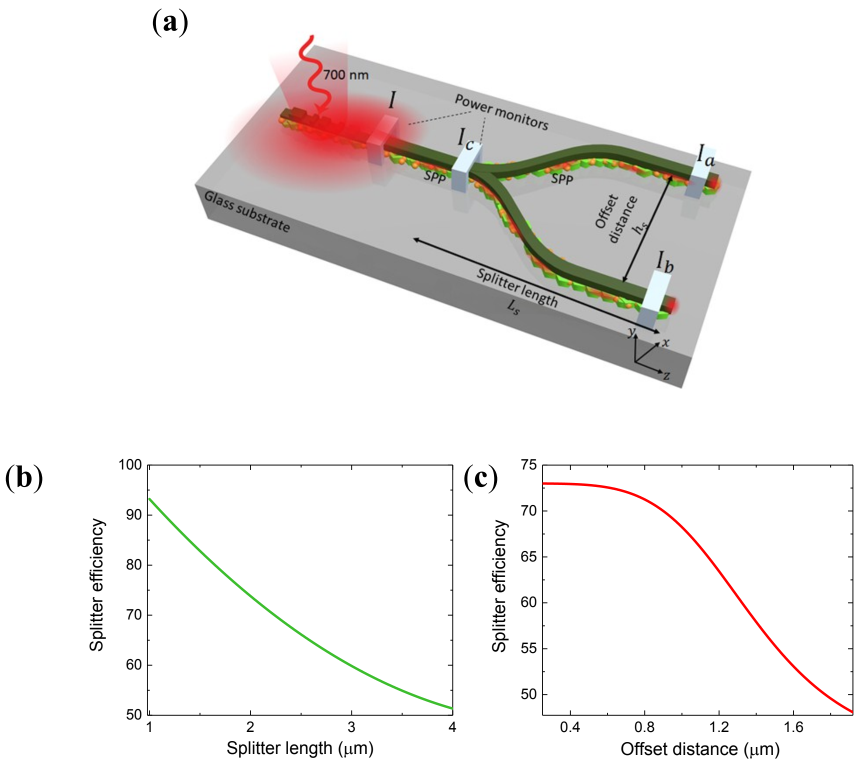

Figure S9: (**a**) Schematic of the plasmonic splitter device composed of two mirrored S-bends of 100 nm x 70 nm of width and thickness, respectively modelled with FDTD simulations at 700 nm operating wavelength. Monitors are used to read the power flow along the studied system. (**b**) Splitter efficiency dependence on the splitter length for a fixed offset distance, (**c**) Dependence of the Splitter efficiency on the offset distance.

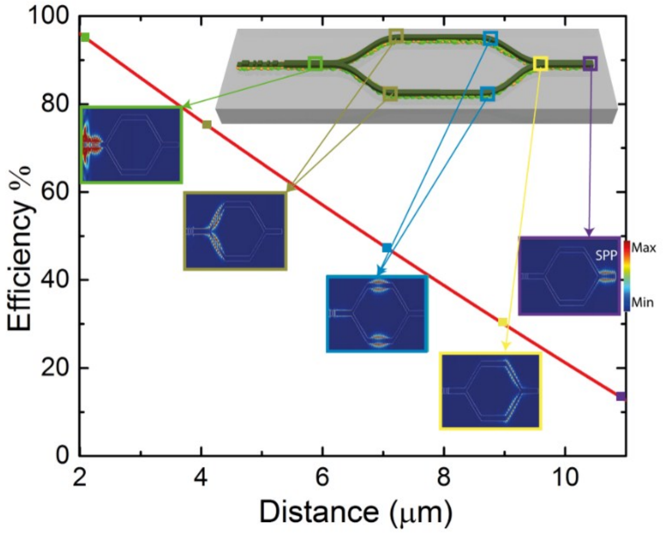

Figure S10: FDTD simulation of bound SPP modes propagating along 11μm length multifunction plasmonic circuitry, coupled with a ND and integrated with optimized grating coupler, showing the SPPs power flow along each part of the circuit.

References

1. P. B. Johnson and R. W. Christy, *Physical Review B*, 1972, **6**, 4370-4379.
